# Small bowel obstruction of barium for gastric cancer screening

**DOI:** 10.1002/jgf2.387

**Published:** 2020-10-12

**Authors:** Maho Adachi‐Katayama, Hiroki Isono, Junya Kashimura

**Affiliations:** ^1^ Department of Medicine Mito Kyodo General Hospital University of Tsukuba Ibaraki Japan; ^2^ Department of General Medicine HITO Medical Center Ehime Japan

## Abstract

We report a case of small bowel obstruction without perforation and peritonitis caused by inspissated barium sulfate for gastric cancer screening and surgically treated 26 hours after the onset of abdominal pain.

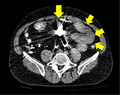

A 55‐year‐old Japanese man presented with abdominal pain. He had a history of robot‐assisted laparoscopic prostatectomy 2 years ago. He did not have constipation. He underwent an upper gastrointestinal series (GIS) at our hospital for cancer screening 1 day previously. He used laxatives and had a small stool. By the afternoon, he was suffering from abdominal pain, abdominal distention, and nausea. The following morning, his abdominal pain had not improved.

On admission, he was mildly ill. His abdomen was soft and distended, with no tenderness, and a normal bowel sound was noted. Laboratory data showed an elevated white blood cell count of 13 400/mm^3^, but were otherwise unremarkable. Abdominal x‐rays showed a small bowel loop and niveau (Figure 1). Abdominal contrast computed tomography revealed a distended small bowel loop, barium accumulation, and small ascites (Figure 2). Cardiovascular hemodynamics were not impaired. He was diagnosed with a small bowel obstruction from inspissated barium sulfate and admitted for observation. An abdominal x‐ray performed 7 hours later did not show the movement of the barium, indicating a high risk of perforation. The decision to operate was taken promptly.

**FIGURE 1:**
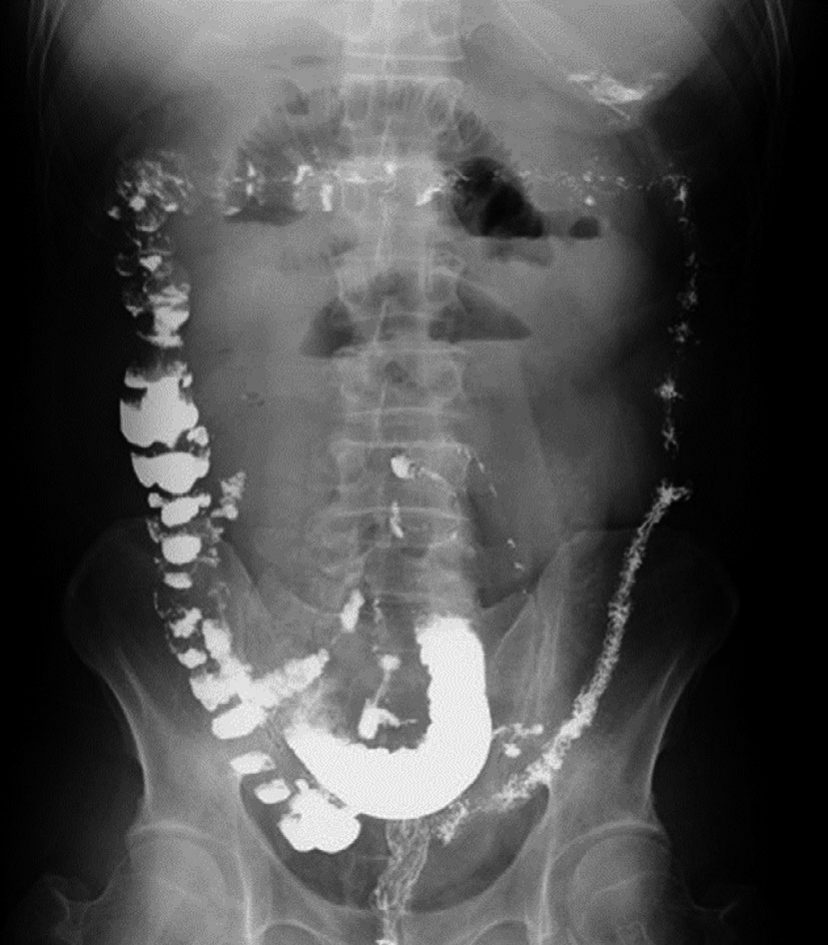
Abdominal x‐ray showing a small bowel loop and niveau

**FIGURE 2:**
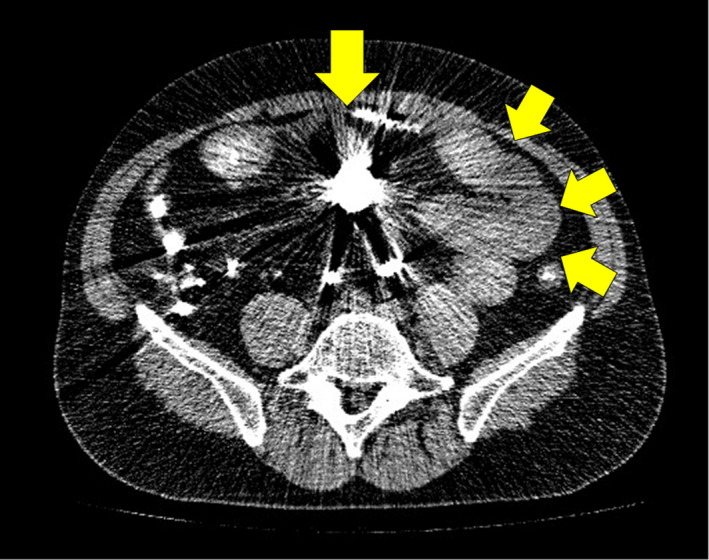
Abdominal CT with contrast material showing distended small bowel loops, barium accumulation (arrow), and small ascites

The intra‐intestinal pressure was reduced by means of an ileus tube prior to the operation. The operation was performed the same day, approximately 26 hours after the initial onset of the abdominal pain. In the operation, the small bowel adhesions were observed in the supraumbilical region. We separated the adhesions and suctioned the barium. We did not perform small intestinal resection because there was no leakage of barium into the abdominal cavity and the serosal surface of the small intestine was normal. The ileus tube was retained. He was discharged 10 days later without perioperative problems.

In Japan, the main procedure for gastric cancer screening is GIS, since being carried out as a national program in 1983. Bowel obstruction and perforations are reported in four and three of 1.1 million patients, respectively. However, bowel obstruction can easily lead to perforation and peritonitis. In addition, the reported mortality rate of barium peritonitis is 29%.^1^

The present patient was surgically managed for a bowel obstruction without perforation caused by inspissated barium, 26 hours after the onset of abdominal pain. Previous cases have shown that the mean day of bowel obstruction was ~3.0, and most patients had perforations when they presented to the hospital. Some cases develop perforation during observation.^2^ Colectomy and colostomy are generally planned for patients with perforation, and the mortality rate of surgery is reportedly 18.2%–20.0%.^2^, ^3^, ^4^ In this case, we first admitted the patient for observation, and when the abdominal x‐ray did not improve after 7 hours, the decision to operate was taken. The operation was successful.

Patients who have a previous history of surgery may develop adhesions and are at risk of bowel obstruction. One study reported that the elderly population comprise a high‐risk group for barium‐induced intestinal obstructions, as they tend to forget to take laxatives after GIS.^5^ Abdominal pain after GIS using barium sulfate should be managed carefully in order to prevent a perforation.

## CONFLICT OF INTEREST

The other authors have stated explicitly that there are no conflicts of interest in connection with this article.

## AUTHORS' CONTRIBUTIONS

MA and JK managed the patient. MA, HI, and JK wrote the manuscript. All authors have approved the final manuscript.

## ETHICAL APPROVAL

The informed consent was obtained to publish this case report.
